# Supplementary material for: Patterns of Intron Gain and Loss in Fungi
Source: PLoS Biol. 2004 Nov 30;2(12):e422. doi: 10.1371/journal.pbio.0020422 (PMC532390; doi:10.1371/journal.pbio.0020422)
Supplement: Table S1 — Also available at http://genes.mit.edu/NielsenEtAl/. (4.3 MB ZIP). [file pbio.0020422.st001.zip › NielsenEtAl/html/1345.html]

AN5021.1.NCU09559.1.MG00195.1.FG04456.1


```
 CLUSTAL W (1.82) Multiple Sequence Alignments - Introns Inserted


Sequence 1: NCU09559.1	713 aa
Sequence 2: MG00195.1	696 aa
Sequence 3: FG04456.1	702 aa
Sequence 4: AN5021.1	667 aa
Alignment Length: 735 aa
Number Identitical Residues: 271 aa
Alignment Score (without introns) 16235


MG00195.1 	-------~----MSQPTRQFSTGASAHRRRQMSTVIEKEGHFGVELT0TLFLGISAVFAD
NCU09559.1	-------~---MAFEKARKFSTGTSVHRKRQMSTLVEKEGHFGPALT0TLYLGISAVFAD
FG04456.1 	-------~--MSSNKPSRKFSTGATSHRKRQMSLMVEKDGHINAPLQ0TLYLGISAVFAD
AN5021.1  	MLPRLRD1NLFDMLGGGSPSPRRASLQQKRRLSTEHSPNSWGAATTG0NLYGGLSTVFEK
          	  .   . .           .  :: :::*::*   . :.        .*: *:*:** .

MG00195.1 	DHTAVVALAIHDTTYLIDFSVKHIVLDDAFKIGEDVISDFVIASVKEYERENFAKFIGAG
NCU09559.1	DHTAVVALAIHDTVYLVDFSVKHIELDDALKMGEDLIAEYVISEVQKYEHENFSKFVGAG
FG04456.1 	DHTAVIALAIHDTVYLNDFSIKHVSLDEEMRQGQDLIADHIINEVETYEHVNFVKFIGAG
AN5021.1  	DK-VIFAVAIRDATYLVDFAQEELPFSESDDLDL-KIGDHMMRHLYKWCDTHLEKMIGLA
          	*: .:.*:**:*:.** **: :.: :.:    .   *.:.::  :  :   :: *::* .

MG00195.1 	LPMTLKYMSPTICSRLWLETDIVPIVIRPDD-----------EHKEKTFWD~AK--RVDE
NCU09559.1	LPTTLKYMSPTLCSRLWLEVDIVPIVMRPDD-----------EHKEATFWD0GPPCPVSS
FG04456.1 	LPVTLKYMSPSLCSRLWLDLDIVPVVLRPDH-----------EAKEKNFWD~VK--RVDE
AN5021.1  	IPKQLAHTCPTICSRLWLELDIIPLVLSDNSKLDFRGEDLQYGFQQSADWE~LR--TLDE
          	:*  * : .*::******: **:*:*:  : . .  ... .   ::   *:      :..

MG00195.1 	QADSMARKCIM2HFGPSLAPLLHVGFRGIVQTDAGFRAPINTIQNNRDVCSAASWETMLT
NCU09559.1	ALKSILTALVR~HFGPSLVPLLQVGFRGIVQTDAGFRAHLTTVQNHKDTCGPATWETTLT
FG04456.1 	QADSMARKCIL2NFGPSLVPHLQVGHRGIVQTDAGFRVHLTNIQNHKDTCSLATWNATQF
AN5021.1  	QAESMARKCVR2LFGPEGTPLLQVGLSGLVQVDTAFHVQLTNKDNYKNSVTANTWKAIEH
          	  .*:    :   ***. .* *:**  *:**.*:.*:. :.. :* ::     :*::   

MG00195.1 	YAKKLRGNKTKVAFFSSTPQGGGVALMRHALVRFSRLMGVDLTW~YV1PKPRPGVFRITK
NCU09559.1	FAKKLRANKLKMAFFSSTPQGGGVALMRHALVRFARLLGVDLTW~YV1PKPRPGVFRITK
FG04456.1 	YANKLREKKTKLAFFSATPQGGGVALMRHALVRLSRLMGVDVTW~YV1PKPRPGVFRITK
AN5021.1  	YADDLRNRKVKLAFFSATPQGGGVALMRHAMVRFAHSIGTDMKW2YV~PKPRPGVFRITK
          	:*..** .* *:****:*************:**::: :*.*:.* ** ************

MG00195.1 	NIHNILQGVSHPDQRISSDEMAAIIDWISDNAQRYWFSEGGPLRPASEGGADVVI0IDDP
NCU09559.1	NIHNILQGVSHPDQRVSAEEKQAIIDWINENASRYWFSEGGPLRAPEEGGADIVV~IDDP
FG04456.1 	NQHNILQGVSHPDQRISDAEKGAISDWIEDNAKRYWLSEGGPLRPPEEGGADIIF0IDDP
AN5021.1  	TNHNILQGVSAPEERLTDENWEQVTNWIQENADRYWLRPGGPLEHPSKGGADVIV~IDDP
          	. ******** *::*::  :   : :**.:**.***:  ****. ..:****::. ****

MG00195.1 	QMPGLIPMIKRLTPDRPVLYRSHIQIRSDLVAISGSPQAEVWDFLWSNIKGADMFISHPI
NCU09559.1	QMPGLIPLIKKYTPNRPVLYRSHIQIRSDLVAKAGSPQADIWDFLWGNIQGADMFISHPI
FG04456.1 	QMPGLIPMIKRLTPDRPVLYRSHIQIRSDLVAIDGSPQNDIWNYLWSNIKEADMFISHPI
AN5021.1  	QMPALIPLAKQKAPERPVVFRSHIQIRSDLVDQDGSPQAECWGTMWKDIQKADIFVSHPV
          	***.***: *: :*:***::***********   **** : *. :* :*: **:*:***:

MG00195.1 	PIFVPHNVPR~EKVVYFPATTDWLDGLNKPLSRWDAGYYGHLYNVAAHSQRQTELNWPAR
NCU09559.1	PSFVPHNVPR~EKVVYLPATTDWLDGLNKHLNHWDSGYYGNLYNNACHSQRMTELNWPAR
FG04456.1 	PKFVPHTVPK~EKVVYLPATTDWIDGLNKHMNKWDTGYYAHIYNTQCRNQRMTELDWPNR
AN5021.1  	KSFVPKIVPR1---IFEKTTLTRED-----------------------------------
          	  ***: **:    ::  :*    *                                   

MG00195.1 	~KYIVQVARFDPAKGIPTVIDSYAEFRRRCEKEG-VTDIPQLVV~CGNGSVDDPDASMIY
NCU09559.1	~KYIIQVARFDPSKGIPTVIDSYAEFRRRCDKAG-ITDVPQLVV~CGNGSVDDPDASLIY
FG04456.1 	1KYIAQVARFDPAKGIPTVIDSYAEFRRRCDEAN-ITEVPQLVV2CGNGSIDDPDGAIIF
AN5021.1  	~EYIVQIARFDPSKGIPHVIESYEKFHQRMQDYCPDKPIPKLLI~CGHGSVDDPDGSIIY
          	 :** *:*****:**** **:** :*::* :.  . . :*:*:: **:**:****.::*:

MG00195.1 	DQTMTQMETHYPHLIKDVSIMRLEPNDQLLNTMIANAHVVLQLSTREGFEVKVSEALHAG
NCU09559.1	DQTMAQLETYYPDLIRDVSVMRLEPNDQVINTLLSNAHVALQLSTREGFEVKVSEALHAG
FG04456.1 	DQTMTQLEDHYPHLLDDVSVMRLDANDQLLNMVIANAHVILQLSTREGFEIKVSEALHAG
AN5021.1  	DLAVRYIEDSIPDLADQICVVRLGPSDQVLNALMSKAKVALQLSTREGFEVKVSEAIHKG
          	* ::  :*   *.*  ::.::** ..**::* ::::*:* **********:*****:* *

MG00195.1 	RPVIATLAGGIPLQVKDGINGFLVQPGDWKTVAGHLISLFTDHELHKKMSHAAATGVSDE
NCU09559.1	RPVIVTNVGGIPLQVKDKVNGFLVAPGDWRAVAGHLMDLFTDDELWKRMHHAARTGVSDE
FG04456.1 	VPVIVSNEGGIPLQVKDNVNGYLVTPGDYKTVAKHLMDLYTDHDLHARMSREAKNGVSDE
AN5021.1  	RPVIATRAGGIPLQVIDKGNGFLVDVGDTDAVAKHLFDLCTDDVLWKKMHEFALAHVCDE
          	 ***.:  ******* *  **:**  **  :** **:.* **. *  :* . *   *.**

MG00195.1 	VGTIGNAIGWYYLASKWNEVGVEKNGKGGLRGDEKWVNDMAREEAGFPYVQNENRLPRHF
NCU09559.1	VGTVGNALAWFYLAAKWTEVGVETSGKGGLKGNEQWVNDMARTEAGYLYTQEENRLPRHF
FG04456.1 	VGTVGNALGWFYLAAKWQELGTNP----GLRGDEKWVNDMAREEAGYPYSEGENRLPRHF
AN5021.1  	VSTVGNSLNWLYLASKLSKGGVIK-------PNERWLNDMAREDAGIPYQEGEDRLKR--
          	*.*:**:: * ***:*  : *.          :*:*:***** :**  * : *:** *  

MG00195.1 	TQVK--------KLPVQTA------
NCU09559.1	TQRKPESESESKDLPIHEKKAEVTA
FG04456.1 	TQRK----EGAQNGKVQEDGNNE--
AN5021.1  	------------ELEVQKMG-----
          	            .  ::
```
